# Supplementary material for: POSS Engineering of Multifunctional Nanoplatforms for Chemo-Mild Photothermal Synergistic Therapy
Source: Int J Mol Sci. 2024 Jan 13;25(2):1012. doi: 10.3390/ijms25021012 (PMC10816201; doi:10.3390/ijms25021012)
Supplement: Supplementary file 1 [file ijms-25-01012-s001.zip › ijms-2804854-supplementary.pdf]

POSS Engineering of Multifunctional Nanoplatforms for Chemo-Mild Photothermal Synergistic Therapy

Supporting Information (6 pages)

1. *Synthetic routes of all compounds* S2
2. *The calculation of photothermal conversion efficiency for* ***POSS-SQ-DOX*** *NPs* S2
3. *Cell culture* S3
4. *Animal model* S3
5. *^{1}H NMR and FTIR of all compounds* S4
6. *UV-Vis and fluorescence of* ***SQ-N*** S5
7. *Contact angles of* ***POSS-SQ-DOX****, POSS-PEG,* ***SQ-N*** *and POSS-SH* S5
8. *Electron microscopic photographs of cell membrane disruption and cell death* *S6*

1. Synthetic routes of all compounds

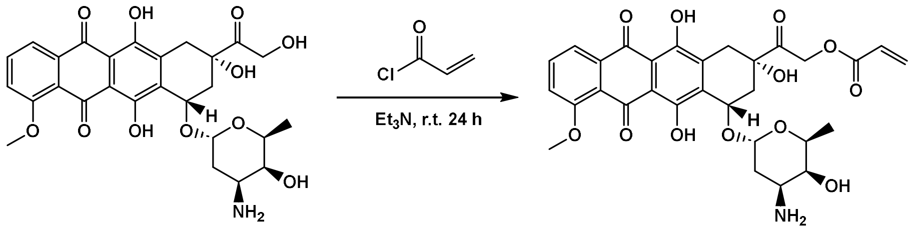

Scheme S1 Synthetic routes of the **DOX-AC**.

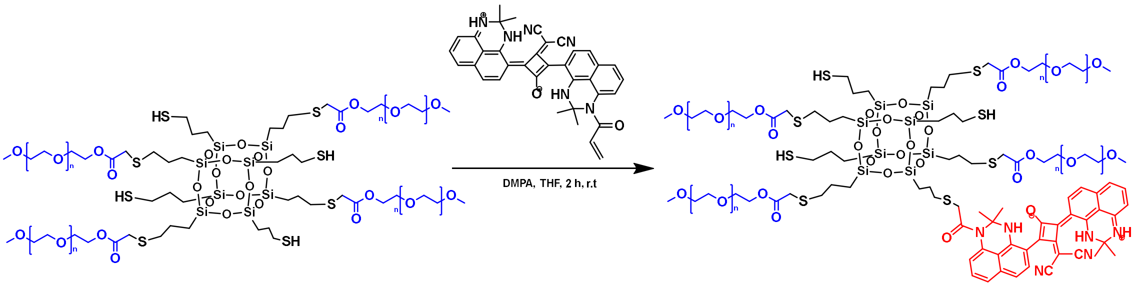

Scheme S2 Synthetic routes of the **POSS-SQ**.

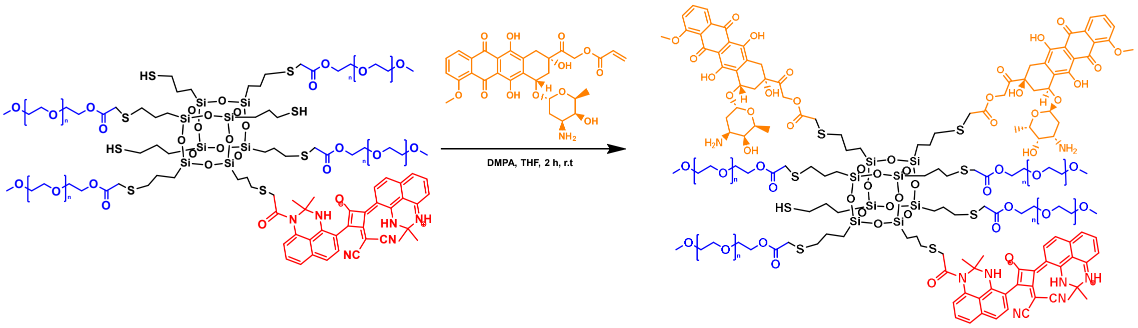

Scheme S3 Synthetic routes of the **POSS-SQ-DOX**.

2. The calculation of photothermal conversion efficiency for ***POSS-SQ-DOX*** *NPs*

The photothermal conversion efficiency (PCE) of photothermal agents was calculated according to the following formula (1):

$$\eta = \frac{hS(T_{Max} - T_{Sur}) - Q_{Dis}}{I(1 - 10^{-A_{808}})} \qquad (1)$$

where h is the heat transfer coefficient, S is the surface area of the quartz plate. T_{Max} (81.1 °C) and T_{Sur} (23.9 °C) are the maximum system and surrounding environment temperatures. I is the laser power (0.5 W/cm^2), and A_{808} is the absorbance of the **POSS-SQ-DOX** NP solution at 808 nm (0.67). Q_{Dis} is the heat dissipated from the light absorbed using a solvent and container with cuvette cell-containing water. hS can be calculated using formula (2):

$$hS = \frac{m_D C_D}{\tau_s} \qquad (2)$$

where m_D and C_D are the mass (0.1 g) and heat capacity (4.2 J/g °C) of water, respectively. τ_s is the time constant of the sample system and can be calculated using formula (3):

$$t = -\tau_s \ln(\theta) \qquad (3)$$

where θ is introduced as a dimensionless parameter in formula (4):

$$\theta = \frac{T - T_{Sur}}{T_{Max} - T_{Sur}} \qquad (4)$$

As a result, τ_s was calculated to be 60.1 s according to formula (3); the photothermal conversion efficiency (η) of the **POSS-SQ-DOX** NPs was determined to be 62.3%.

3. *Cell culture*

L929 and cervical cancer (HeLa) cell lines were cultured in Dulbecco's modified Eagle's medium (DMEM) containing 10% fetal bovine serum (FBS) and a 1% dual antibody (penicillin and streptomycin), which were then grown under humidified air containing 5% CO_2 at 37 ˚C.

4. *Animal model*

All the animal experiments in this work were carried out under the guidelines of the Shanghai Jiao Tong University Institutional Animal Care and Use Committee. To establish the HeLa tumor-bearing mouse model, HeLa cancer cells (approximately 5×10^6 cells suspended in 100 μL of PBS) were subcutaneously injected into the right flanks of mice. After about a week, the tumor volumes reached 120 mm^3, and the mice were used for in vivo photothermal therapy studies subsequently, and various treatments were applied to the tumor sites.

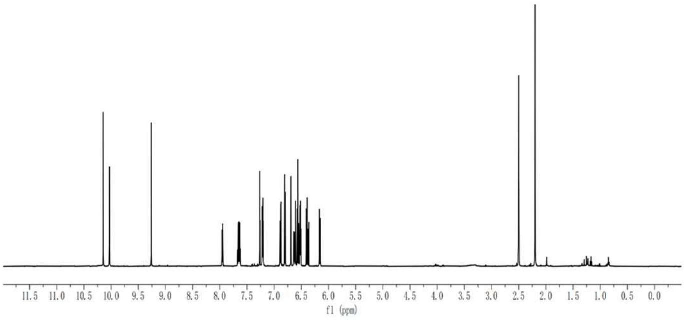

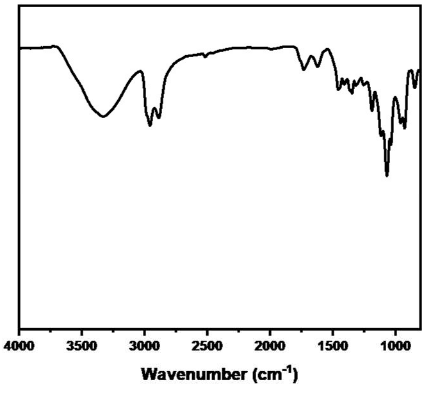

Figure. S1 **a** ^{1}H NMR spectrum (600 MHz, DMSO) and **b** FTIR of **DOX-AC**.

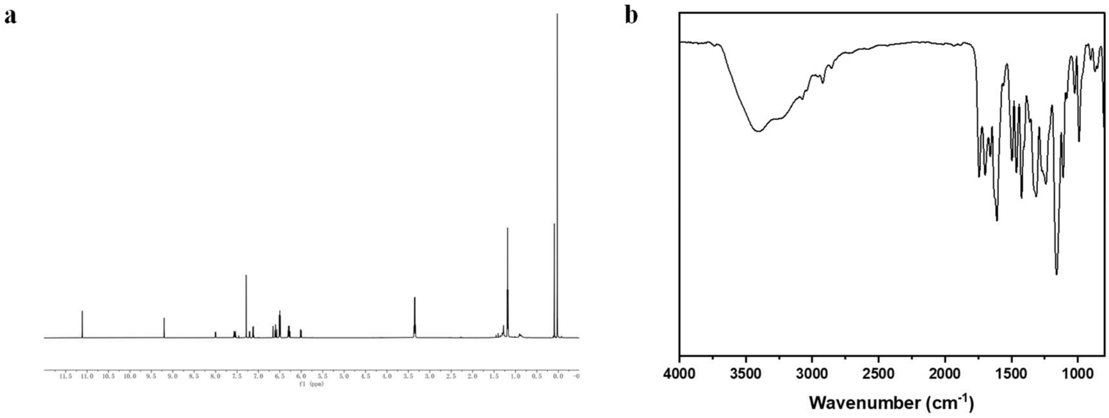

Figure. S2 **a** ^{1}H NMR spectrum (600 MHz, DMSO) and **b** FTIR of **POSS-SQ**.

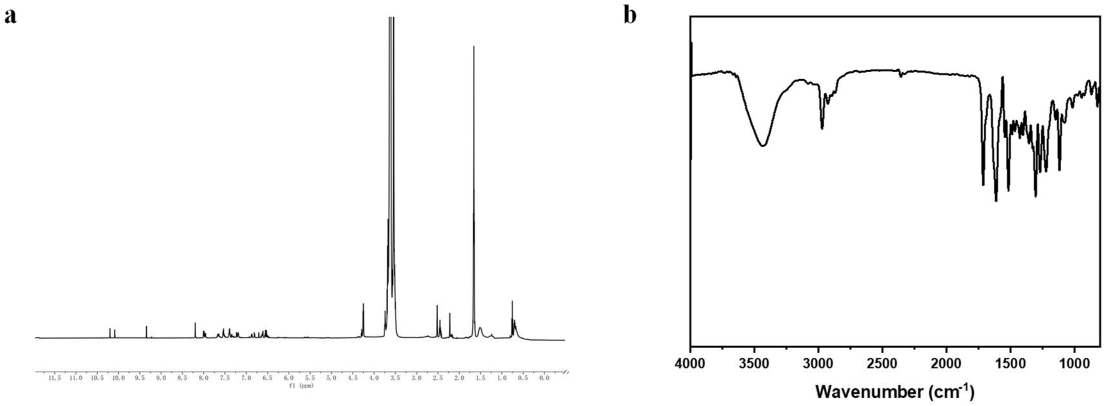

Figure. S3 **a** ^{1}H NMR spectrum (600 MHz, DMSO) and **b** FTIR of **POSS-SQ-DOX**.

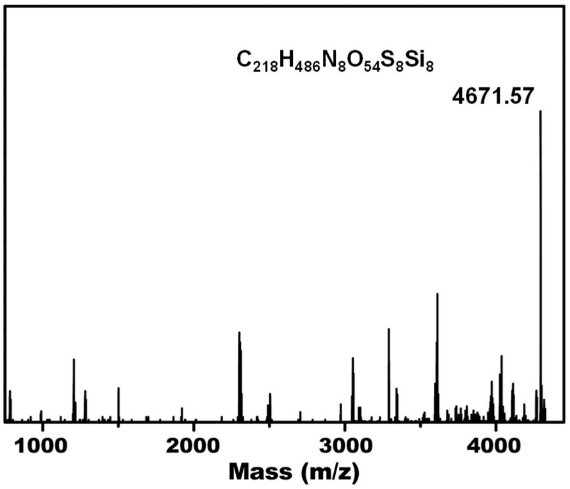

Figure. S4 ESI-MS spectrum of **POSS-SQ-DOX**.

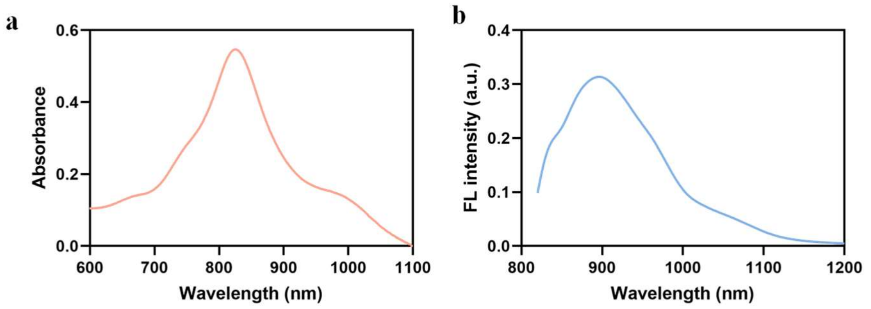

Figure. S5 **a)** UV-vis absorption and **b)** fluorescence spectra of **SQ-N** (25 μg/mL) in aqueous solution

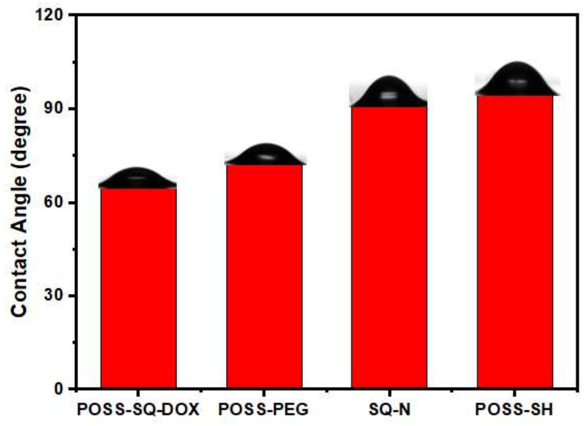

Figure. S6 Contact angles of **POSS-SQ-DOX** NPs, POSS-PEG, **SQ-N** and POSS-SH.

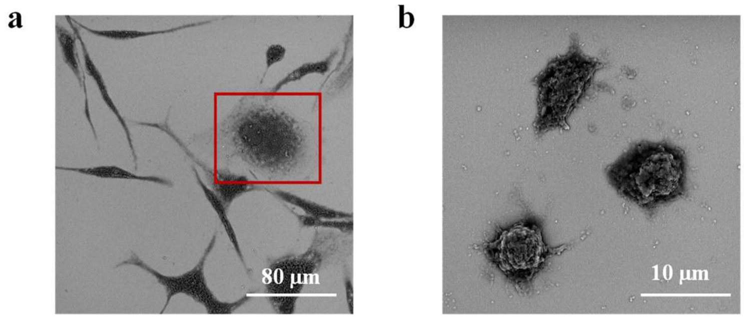

Figure S7. Electron microscopic photographs of **a)** cell membrane disruption and **b)** cell death.
